# 31115 Perimetric resilience in the retina: spatial distribution of preserved peripheral visual field loci in retinitis pigmentosa

**DOI:** 10.1017/cts.2021.468

**Published:** 2021-03-30

**Authors:** Hursuong Vongsachang, Tapan Patel, Xiangrong Kong, Mandeep S. Singh

**Affiliations:** Wilmer Eye Institute

## Abstract

ABSTRACT IMPACT: We developed a novel semi-automated computational platform to quantify spatial characteristics of preserved peripheral visual field loci in retinitis pigmentosa. This work may inform future research on therapies to prevent visual field loss in retinitis pigmentosa and related diseases. OBJECTIVES/GOALS: Retinitis pigmentosa (RP) causes progressive and severe peripheral visual field (pVF) loss in some but not all patients. The characteristics of pVF in RP are incompletely understood. We developed a novel semi-automated computational platform to quantify the spatial characteristics of pVF. METHODS/STUDY POPULATION: We analyzed preserved pVF size and location in both eyes of RP patients using the Goldmann V4e isopter. We developed a custom algorithm in MATLAB to align and average the binary V4e isopter segmentations, and generated a two-dimensional probability map of the spatial distribution of preserved pVF loci along the radial and circumferential dimensions. To adjust for disease duration, cases were categorized by the time from self-reported symptom onset. Probability maps of pVF preservation were generated for categories of disease duration using unsupervised K-means clustering. Analyzing cases with longitudinal data, we identified loci of stable pVF over time. RESULTS/ANTICIPATED RESULTS: A total of 152 patients were included (N=304 eyes). The mean age was 46.7 years and 49.3% were male. Disease duration was categorized as <20 years (N=72, 47.4%), 20-40 years (N=60, 39.5%), or >40 years (N=20, 13.2%). Longitudinal data (3.2 -5.7 years of follow-up) were available in 65 patients (42.8%). Probability plots of preserved pVF loci in the cross-sectional dataset showed that the median percentage of preserved pVF loci were located between 50 ºand 80 ºeccentricity and between the 30 ºto 50 ºmeridians, with highly concordant inter-ocular symmetry. Probability plots in the longitudinal dataset showed that inferotemporal pVF loci were most likely to be preserved over time. DISCUSSION/SIGNIFICANCE OF FINDINGS: Semi-automated quantification of pVF loci is a useful platform to analyze spatial characteristics of the visual field in RP. Certain portions of the pVF may be relatively resistant to functional decline. Understanding the molecular basis of pVF resilience will inform further research on RP therapy.

